# Seroepidemiology of bovine herpesvirus‐1 in goats in south‐western Iran

**DOI:** 10.1002/vms3.1574

**Published:** 2024-08-23

**Authors:** Mahdi Pourmahdi Borujeni, Amir Hossein Abbasi, Mohammad Rahim Haji Hajikolaei, Masoud Reza Seifi Abad Shapouri

**Affiliations:** ^1^ Department of Food Hygiene, Faculty of Veterinary Medicine Shahid Chamran University of Ahvaz Ahvaz Iran; ^2^ Department of Clinical Sciences, Faculty of Veterinary Medicine Shahid Chamran University of Ahvaz Ahvaz Iran; ^3^ Department of Pathobiology, Faculty of Veterinary Medicine Shahid Chamran University of Ahvaz Ahvaz Iran

**Keywords:** bovine herpesvirus type 1, epidemiology, goat, host, prevalence, reservoir

## Abstract

**Background:**

Widely regarded as one of the chief causes of diseases in cattle population, bovine herpesvirus‐1 (BoHV‐1) has the potential to infect sheep and goat, making them potential reservoirs or hosts for this virus. Thus, preventive measures against BoHV‐1 in cattle should not overlook the ability of this virus to infect other animals.

**Aims:**

Therefore, the focal point of this study was to ascertain the seroprevalence of BoHV‐1 in 300 healthy goats, the relationship between host and the environmental determinants of infection, and the contributing role of goats in the epidemiology of the BoHV‐1.

**Materials & Methods:**

In order to pinpoint the existing antibodies to BoHV‐1, the obtained sera were analyzed by Virus Neutralization test.

**Results:**

According to this test, the seroprevalence of BoHV‐1 appeared to be 64.33% in southwestern Iran. What logistic regression disclosed was that the odds ratio between age and infection with BoHV‐1 was 0.83 (*p* = 0.01), representing a decrease of 17% as goats grew one year older. In addition, females manifested a higher relative frequency of infection compared to males, with the odds of infection in female goats being registered at 1.88, compared to those in males (*p* = 0.2). Moreover, contrasted with goats lacking any history of abortion, those with a history of abortion featured 1.1 as the odds ratio (*p* = 0.87). The seroprevalence in Hendijan, Ahvaz, Shushtar and Dasht e Azadegan was detected to stand at 73.24, 71.30, 55.56 and 47.06 percent, respectively, with 6% of fluctuation in the infection rates being attributed to various geographical locations under the scrutiny of this study (*p* = 0.003).

**Discussion and Conclusion:**

Having attested the marked seroprevalence of BoHV‐1, the definitive role of goats in the epidemiology of this virus as a secondary host or reservoir was confirmed by the present study, necessitating the strict monitoring of BoHV‐1 in goats by animal health authorities in areas where BoHV‐1 abounds in cattle.

## Graphical abstract

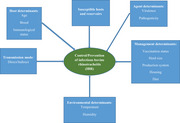

## 1 INTRODUCTION

Infectious bovine rhinotracheitis (IBR) is a highly contagious disease infecting domestic and wild ruminants, especially cattle. The heavy economic losses inflicted on cattle breeding industry arise from weight loss and reduced milk production, fertility disorders, abortion, and respiratory disorders; thus, radical measures are being taken to prevent or even eradicate this disease in different countries (Constable et al., 2017; Muylkens et al., 2007; Nettleton & Russell, 2017). Despite being successfully eradicated in some European countries, due to the eccentric nature of this disease, covert clinical symptoms, and the complications of laboratory diagnosis, this disease is still prevalent in many parts of the world (Constable et al., 2017; Majumder et al., 2015; Nettleton & Russell, 2017; Waldeck et al., 2021). The causative agent of IBR is bovine herpesvirus type 1 (BHV‐1 or, more recently, BoHV‐1). This virus finds its way to the host's body through mouth or genital tract, and its infection rate has been reported to be 37%–67% in beef cattle and 25.9–64.1 in dairy cattle; however, factors, such as age, vaccination status, season, herd size, production system (dairy or meat) and herd management, are considered major risk factors of this disease (Constable et al., 2017; Nardelli et al., 2008; Nettleton & Russell, 2017; Raaperi et al., 2010; Segura‐Correa et al., 2010; Waldeck et al., 2021; Woodbine et al., 2009). Based on the genetic analysis of BoHV‐1, which belongs to the Herpesviridae family with a subfamily of Alphaherpesvirinae and *Varicellovirus* genus, it can be divided into two main subtypes, including BoHV‐1.1 and BoHV‐1.2 (subdivided into 1.2a and 1.2b) (Muylkens et al., 2007; Nettleton & Russell, 2017; Ramakrishnan et al., 2018). In addition, BoHV‐1 is associated with and possesses cross‐reactions with some herpesviruses related to domestic and wild ruminants, such as BoHV‐5, caprine herpesvirus type 1 (CpHV‐1), cervid herpesvirus types 1 and 2, bubaline herpesvirus type 1 and elk herpesvirus. By way of example, CpHV‐1 causes digestive disorders in young goats, whereas abortion and fertility disorders occur in adults. More importantly, CpHV‐1 and BoHV‐1 both can transmit to other hosts (heterologous host); subsequently, that infected animal plays the role of reservoir or secondary host for the primary one (Six et al., 2001). Furthermore, Tolari et al. (1990) reported the isolation of BoHV‐1 from an outbreak of meningoencephalitis in goats and reactivations of infection in a naturally infected goat belonging to the same herd by injection of dexamethasone. Thus, considering the possible diversity and overlapping genetic as well as host diversity, the control and prevention of BoHV‐1 become increasingly complicated (Constable et al., 2017; Yeşilbağ et al., 2003). Given the fact that sheep and goats are less susceptible to the virus, they can act as possible sources of BoHV‐1 infection for cattle (Gür et al., 2019; Pourmahdi Borujeni et al., 2020; Thiry et al., 2006; Tolari et al., 1990; Yilmaz & Coskun, 2016). It has been disclosed that this virus can remain hidden in goats (the trigeminal ganglia of goat was identified as one site of latency) and manifest itself as mild symptoms of pneumonia and conjunctivitis during acute infection (Six et al., 2001; Thirty et al., 2006).

Because the ultimate objective of investigating the epidemiology of a disease is its prevention, control and, more obviously, the eradication of the disease, sensitive hosts should be in the spotlight. In view of the prevalence of traditional breeding of livestock in Khuzestan province, south‐western Iran, and raising them simultaneously, the significant rate of infected cattle in this region with BoHV‐1 (Adeli et al., 2017), and the confirmed role of sheep as a reservoir (Pourmahdi Borujeni et al., 2020), the current study aimed at determining the infection level of goats with BoHV‐1 virus in this province as well as their possible role as another reservoir of this virus in this region.

## 2 MATERIALS AND METHODS

### 2.1 Study area

The current cross‐sectional study was conducted in the tropical province of Khuzestan, situated in the southwest of Iran, the climate of which varies from arid to humid. Interestingly, although the northern regions experience cold weather, tropical climate dominates the southern parts. In this province, more than 300,000 cattle, 2.6 million sheep and 1.2 million goats are kept, the breeding of which is mainly traditional and, to some extent, semi‐industrial. Goats are primarily raised for their milk and meat, usually kept mixed with sheep and cattle and, to some extent, with buffaloes (Statistical Centre of Iran, 2017).

### 2.2 Sample size and characteristics of the samples under review

The sample size for this research was determined using the following formula (Thrusfield et al., 2018). The minimum sample size for 95% confidence level, 28% expected prevalence (Pourmahdi Borujeni et al., 2020) and 5.5% precision was 256 goats. However, in this study, 300 goats from 15 herds were scrutinized (at least 10% of goats from each were randomly selected):

$$n=\frac{{\left(Z_{1-\frac{a}{2}}\right)}^{2}P\left(1-P\right)}{{\left(d\right)}^{2}}$$

To this end, blood samples were randomly collected from 300 clinically healthy goats with at least 6 months of age in cooperation with the veterinary networks of Ahvaz, Handijan, Dasht‐e Azadegan and Shushtar cities. A 10 mL blood sample was obtained from the jugular vein after disinfecting the site by VENOJECT (EXPILAB, Gel & Clot Activator). Furthermore, all data related to various features of goats, including age (through questioning and observation of teeth), sex (via observation), history of abortion (via inquiry) as well as geographical region, were gathered. The mean and standard deviation of the age of goats were 3.32 and 1.73 years, with the relative frequency of female and male genders being 94% and 6%, respectively. Next, the selected animals were divided into three age groups (≤1: young, 2–3: subadult and ≥4 years old: adult). The history of abortion was recorded in 5.76% of adult female goats. The absolute frequency of samples collected in Ahvaz, Handijan, Azadgan and Shushtar was 115, 71, 51 and 63 heads, respectively.

### 2.3 Serological analysis

Virus neutralization (VN) test as well as the guidelines presented in the WOAH (World Organisation for Animal Health) (2024) guide were used to check the presence of antibodies against BoHV‐1 virus in the sera. For this purpose, serum samples were first heated at 56°C for 30 min to inactivate the complement. Then, 50 µL of each undiluted sera were transferred into two wells next to each other in a 96‐well cell culture plate. In the next step, 50 µL of BoHV‐1 virus suspension (this virus is a field strain isolated by Razi Vaccine & Serum Research Institute, Hesarak, Karaj, Alborz, Iran, which is used as a standard isolate in diagnosis and research [Maghami et al., 2023; Shirvani et al., 2011]), containing 150 TCID50, was added to each well. After an overnight incubation at 37°C with 5% CO_2_, 100 µL of RBK cell suspension (containing about 10,000–15,000 cells) in RPMI culture medium containing 2% foetal bovine serum was added to each well, and the plate was incubated at 37°C with 5% CO_2_ for 3–5 days. The wells of serum samples were compared daily with the control wells of virus and cells in terms of cytopathological effects (CPE). The wells showing CPE were considered negative, and the absence of CPE was considered positive.

### 2.4 Statistical analysis

SPSS (Version 16.0; SPSS Inc.) was used for the statistical analysis of the data, whereas the Chi‐square test and logistic regression were deployed to determine the relationship among age, sex, history of abortion as well as the geographic location with infection. For every single potential related factor, bivariate logistic regression models were made compatible with the data. However, to boost precision, a multivariate logistic regression model, encompassing a backward and stepwise algorithm, was utilized to further pinpoint the risk factors in conjunction with BoHV‐1 (*p *≤ 0.3) in bivariate regression. The Hosmer and Lemeshow tests verified the goodness of fit of the model. The possible correlation between species and infection was detected via the contingency coefficient value; meanwhile, confidence interval was calculated by Wald's method (Thrusfield et al., 2018). Statistically significant differences (*p* ≤ 0.05) were taken into account.

## 3 RESULTS

The seroprevalence rate of BoHV‐1 infection in the studied goats was 64.33% (95%CI: 58.91%–69.75%), and the entire herds under investigation were seropositive. The statistical analyses divulged a relationship between infection and age orders, that is, diminishing infection accompanied aging (*χ* ^2^ = 6.25, df = 2, *p* = 0.04) (Table 1). Furthermore, univariate logistic regression revealed 0.83 (95%CI: 0.73–0.96) as the odds ratio between the age, based on year and infection, meaning that a 17% reduction in infection coincided with 1 year ageing. In addition, the age factor served as a justification for 3.1% of fluctuation in infection (Table 1). With regard to sex, the prevalence rates of BoHV‐1 infection in female and male goats stood at 65.25% and 50.0%, respectively, with the Chi square test not revealing a marked disparity between these sex groups (*χ* ^2^ = 1.11, df = 1, *p* = 0.29). Females had 1.88‐fold increased odds of being infected compared to their male counterparts. Furthermore, the factor of gender gave a justification for 0.8% of fluctuation in infection rate (Table 1). No remarkable difference was noted between BoHV‐1 infection and a history of abortion (*χ* ^2^ = 0.01, df = 1, *p* = 1). Standing in contrast to those with no history of abortion, the odds ratio in those featuring a history of abortion recorded 1.1, with 0.1% of fluctuation in infection rate being accounted for by this factor (Table 1). As shown in Table 1, different counties demonstrated varying degrees of infection. Hendijan and Ahvaz with 73.24% and 71.3%, and Dasht‐e Azadegan with 47.06% prevalence rates represented the highest and lowest rates of infection, respectively. As might be expected, notable differences were detected between infection and geographical location (*χ* ^2^ = 13.64, df = 3, *p* = 0.003), as 6.0% of fluctuation in infection was justified by geographical location (Table 1). What is more, an array of factors, namely age, sex, history of abortion and geographical location, served to justify 8% of fluctuation in infection as disclosed by multivariate logistic regression. According to backward stepwise multivariate logistic regression among age, sex and geographical location (*p* ≤ 0.3), it was solely the geographical location that exerted a determining impact on infection (Hosmer & Lemeshow test: *χ* ^2^ = 0, df = 4, *p* = 1).

**TABLE 1:** Seroprevalence of bovine herpesvirus‐1 (BoHV‐1) infection in goats in the southwest of Iran based on age, sex, history of abortion and geographical determinants.

Category	Groups	Point prevalence (%) (positive/sample)	Interval prevalence (95%)	OR (95% CI)	*p*‐Value
**Age**	Young animals^a^	79.17 (38/48)	67.68–90.66	2.65 (1.22–5.78)	0.014
	Sub‐adult animals**^ab^**	64.23 (79/123)	55.76–72.7	1.25 (0.75–2.08)	0.39
	Adult animals^b^	58.91 (76/129)	50.42–67.4	1	–
**Sex**	Female^a^	65.25 (184/282)	59.69–70.81	1.88 (0.72–4.88)	0.2
	Male^a^	50.0 (9/18)	26.9–73.1	1	–
**Abortion**	Delivered normally^a^	62.01 (142/229)	55.72–68.3	1	–
	History of recently aborted^a^	64.29 (9/14)	39.19–89.39	1.1 (0.36–3.4)	0.87
**Geographical location**	Hendijan^a^	73.24 (52/71)	62.94–83.54	3.08 (1.44–6.59)	0.004
	Ahvaz^ab^	71.3 (82/115)	63.03–79.57	2.8 (1.41–5.53)	0.003
	Shushtar^bc^	55.56 (35/63)	43.29–67.83	1.41 (0.67–2.95)	0.36
	Dasht‐e Azadegan^c^	47.06 (24/51)	33.36–60.76	1	–

*Note*: Different lowercase letters in each variable represent a significant difference.

## 4 DISCUSSION

Although it was 70 years ago when BoHV‐1 was first identified, the virus is regarded as a globally significant pathogen. As regards the diverse potentials of this virus to hide, re‐activate and easily transmit among animals kept in high density, it can easily linger in the cattle population, exerting unwelcome effects on their health and productivity. One of the efficacious factors in controlling and especially eradicating an infectious disease is the identification of its sensitive hosts; it is possible for a species or multiple species to act as a secondary host or a reservoir for the survival of a pathogen in a particular region, facilitating its spread to the primary host through interspecies transmission and consequently, triggering the disease. BoVH‐1 is not an exception to this rule, and it is believed that although cattle are the primary hosts of this virus, sheep and goats can also be considered potential sources of the virus for cattle. Based on this hypothesis, the previous studies had also confirmed the possibility of virus transmission among cattle, sheep and goats (Gür et al., 2019; Pourmahdi Borujeni et al., 2020; Wentink et al., 1993); likewise, the experimental studies conducted by Wafula et al. (1985), Shankar and Yadav (1987) and Hage et al. (1997) have affirmed the possibility of infection with BoHV‐1 in goats and sheep.

The present study, the first to have been conducted in Iran, disclosed a significantly high seroprevalence of BoHV‐1 in goats in the south‐western part of Iran (approximately 65%), which was in line with the level of infection in cattle (48.7%) and sheep (28.4%), all indicating the circulation of this virus in the goat population of this region, and making it the chief culprit for the epidemiology of the disease as a secondary host or reservoir (Adeli et al., 2017; Pourmahdi Borujeni et al., 2020). It is worth noting that after a natural infection with BoHV‐1, the animal becomes permanently seropositive (Rüsch et al., 1981). Given the extensive traditional breeding in this province and the intimate contact of cattle, sheep and goats, such remarkable prevalence is justified. Of course, it should not be overlooked that part of this high percentage of infection might be due to CpHV‐1, the major contributor to digestive disorders in young goats and abortion and fertility disorders in adults; nevertheless, because the goats under investigation did not feature any symptoms of sickness, this issue is ruled out to some extent. What is more, less than 6% of the randomly selected goats in this research had a history of abortion in the past. In addition, it has been revealed that each of the CpHV‐1 and BoHV‐1 viruses can cause disease in the primary host but subclinical infection in the secondary host, therefore assuming the role of a reservoir for primary host (Constable et al., 2017; Yeşilbağ et al., 2003). Although the study of Suavet et al. (2016) showed that the goat serum infected with CpHV‐1 can partially neutralize BoHV‐1, the serum of calves experimentally infected with CpHV‐1 (as a heterologous host of this virus) fails to neutralize BoHV‐1. Owing to the fact that goats are also heterologous for this virus, the neutralizing activity of the serum of goats in this study against BoHV‐1 may be more attributed to their infection with BoHV‐1 than CpHV‐1. It should also be emphasized that, so far, there has been no documented report of the frequency of CpHV‐1 in Iranian goats based on a systematic review of electronic databases. Nevertheless, it is recommended that, for solid evidence to be attained, the serum samples of goats be evaluated simultaneously for CpHV‐1 and BoHV‐1 viruses by means of neutralization and titration methods. The relative frequency of BoHV‐1 serum infection in other parts of Iran has been reported to be 7.1%–72% in cattle and 10.7% in sheep (Badiei et al., 2010; Ezzi et al., 2013; Ghaemmaghami et al., 2013; Hashemi et al., 2022; Hashemi Tabar et al., 2009; Kargar Moakhar et al., 2001; Raoofi et al., 2004; Sakhaee et al., 2009; Shirvani et al., 2012). Gür et al. (2019) reported the level of infection in cattle, sheep, and goats to be, respectively, 32.3%, 0.09% and 29.9% in the western neighbour of Iran, Turkey, which indicated a significant correlation between the level of infection in cattle and goats rather than sheep. In other studies conducted in this country, the serum infection rates of sheep and goats were recorded at 1.4%–2.27% and 3.3%–5.2%, respectively (Albayrak et al., 2007; Baydin & Dağalp, 2017; Cabalar & Özgünlük, 2014; Yazici et al., 2021; Yilmaz & Coskun, 2016). BoHV‐1 seroprevalence in goats and sheep has been 27.6% and 23.8% in Egypt, 2.4% and 3.4% in Bulgaria, 29.5% and 47% in Hungary and 13.7% only in goats in South Korea (Kálmán & Egyed, 2005; Mahmoud & Ahmed, 2009; Rusenova & Bochev, 2009; Yang et al., 2008). Interestingly, in Canada, it is 10.8% in sheep (Elazhary et al., 1984) and 0% in sheep and goats (Lamontagne et al., 1985). The percentage of relative frequency in Zaire and Chile sheep was 8% (Celedón et al., 2001; Jetteur et al., 1990), whereas in Japan and Brazil, it was recorded at 0% (Giangaspero et al., 2013; Gonçalves et al., 2011). As it is readily apparent, there are marked variations in the frequency of BoHV‐1 infection among different countries, and even within a country between different regions, which can be attributed to various determinants such as management (type of husbandry, keeping sheep and goats in mixed‐species groups simultaneously with cattle, and the rate of infection in cattle), and climate conditions. What the findings have revealed is that BoHV‐1 can survive for 50 days at 21°C, 10 days at 37°C and 21 min at 56°C (Constable et al., 2017). In addition to the above‐mentioned factors, other elements also merit attention, such as the sampling method (random or non‐random) and the type of test used for antibody detection (VN test, ELISA and immunofluorescence). The VN test, which quantitatively determines the inhibitory effects of specific antibodies on virus replication in cell culture, is considered a standard test for detecting BoHV‐1, providing a benchmark for other assessment methods. Furthermore, the sensitivity and specificity of this test for serum detection of BoHV‐1 have been declared as 94%–98% and about 96%, respectively (Lucas et al., 1986). However, not only is this method time‐consuming (taking 5–6 days), but the necessity of proper laboratory equipment and experienced experts complicates it even further (Constable et al., 2017). Comparing the results of the present research with those of the previous investigations conducted in this area, it was divulged that the infection in goats is significantly higher than in sheep (2.3 times) (*p* < 0.001). In this regard, goats have been found to be more vulnerable to natural and experimental contamination compared to sheep (Gür et al., 2019; Mahmoud & Ahmed, 2009; Wafula et al., 1985; Yang et al., 2008; Yeşilbağ & Güngör, 2009).

In this study, there was no significant relationship between age and infection, although the chance of infection decreased with age; thus, apparently, due to the latent form of herpesviruses, most cases of infection manifest themselves in early stages of life, following direct and indirect contact with the infected animals; in other words, in line with ageing, no noticeable changes in the frequency of infection are observed. Therefore, it may be due to the latent nature of the virus or decrease in antibody titre with age. Although it is also possible to develop a latent infection at a younger age, the lowering of the antibody titre happens later, so the antibody is better detectable. Consistent with the current research, Badiei et al. (2010) in cattle and Pourmahdi Borujeni et al. (2020) in sheep did not observe a significant relationship between age groups and infection; on the contrary, Msolla et al. (1981), McDermott et al. (1997), Boelaert et al. (2005), Kampa et al. (2009), Guarino et al. (2008), Woodbine et al. (2009), Raaperi et al. (2010), Carbonero et al. (2011), Bahari et al. (2013) and Adeli et al. (2017) regarded age as a determining factor associated with BoHV‐1 positive cases in cattle.

In this study, no marked relationship between infection and gender was noted, which was compatible with the findings of Raoofi et al. (2004), Adeli et al. (2017) and Pourmahdi Borujeni et al. (2020). Contrary to this study, Saravanajayam et al. (2015) disclosed the higher prevalence of IBR in females than in males in their study. Ostensibly, BoHV‐1 virus can infect both sexes, not showing any inclination for either, thus negating the possible role of hormonal and genetic differences between the two sexes on the level of infection; hence, the reported discrepancies attributed to these sexes are invalid and arise from confounders such as age and management. Performing a multivariate analysis can control such confounding factors, ruling out the role of gender as a factor influencing infection in this study. In the present study, the relative frequency of BoHV‐1 infection in goats with and without a history of abortion was not meaningfully different. This finding was in alignment with that attained by Raoofi et al. (2004), Adeli et al. (2017) and Pourmahdi Borujeni et al. (2020); however, Biuk‐Rudan et al. (1999) and Tuncer‐Göktuna et al. (2016) reported a meaningful correlation between BoHV‐1 infection and reproductive disorders in dairy cows. Interpreting the association between abortion and BoHV‐1 sero contamination can be challenging because abortion occurs long after BoHV‐1 infection; meanwhile, during abortion, the amount of antibody may diminish so substantially that conventional serological methods, such as neutralization of the virus and ELISA, may fail to detect; what is more, the involvement of other possible factors related to abortion should not be disregarded. Despite this, one should not neglect the fact that there may be an inverse relationship between abortion and BoHV‐1 infection; in other words, aborted animals might lack anti‐BoHV‐1 antibodies, whereas the pregnant animals might possess them. Therefore, isolating the virus from aborted fetuses to determine the cause of abortion can be more productive than attributing the root cause of abortion to BoHV‐1 infection.

According to the present study, the relationship between geographical location and infection was marked, with the majority of positive cases arising from Handijan and Ahvaz. In the previous study, the prevalence of infection in cattle was reported as 93.48%, 77.27%, 56.67% and 20.69% in Ahvaz, Shushtar, Dasht‐e Azadegan and Handijan, respectively (Adeli et al., 2017), and the contingency coefficient between cattle and goat infection in the sample cities varied from 0.1 to 0.46, being meaningful in Handijan, Ahvaz and Shushtar. In terms of the relationship between infection and geographical area, the results of the current study aligned with those of Adeli et al. (2017) and Pourmahdi Borujeni et al. (2020). However, in cities where the rate of infection was at peak, the discrepancies were even more evident; thus, on the basis of the study carried out by Adeli et al. (2017) on cattle, Behbahan featured the highest rate, whereas Ahvaz and Shushtar indicated the least; however, in the study by Pourmahdi Borujeni et al. (2020) on sheep, Shushtar and Masjed Soleyman recorded the highest rate of infection, with Behbahan, Handijan and Ahvaz being the least infected cities. Although the cities of Khuzestan province vary in terms of weather conditions, BoHV‐1 has been found to be relatively resistant to environmental factors; that is, it can survive for a month in low temperatures (4°C) and high humidity (more than 90%), and in hot areas, the survival of this virus has been registered to range from 5 to 13 days (Constable et al., 2017). Therefore, presumably, this virus is located in the same host in each city of this province. However, the role of other influential factors such as management should not be underestimated. Furthermore, the proportion of BoHV‐1 infection in a herd or region may change over time due to changes in influencing factors. The BoHV‐1 infection in cattle of this area was investigated 7 years ago, and during these years, it may have changed, decreased or increased. The herd size of cattle and goats in the cities of this province has changed during these years. It is obvious that BoHV‐1 virus circulates in high‐density herds due to effective contact for transmission at a higher frequency. Therefore, the reported prevalence of BoHV‐1 in goats (present study) is different from cattle (last study) in this province.

## 5 CONCLUSION

What this research divulged was that quite comparable to cattle population, BoHV‐1 virus is in circulation in the goat population of Khuzestan province with high frequency, which highlights the role of goat in the epidemiology of this viral disease as a reservoir or secondary host. So, although the main source of the BoHV‐1 is cattle, the contact between goats and cattle could provide added opportunity for further onward transmission and spread of this virus through respiratory secretions (aerosol transmission). Therefore, health policymakers should take this issue into account and incorporate it as an intervention factor in IBR control and prevention programmes.

## AUTHOR CONTRIBUTIONS

**Mahdi Pourmahdi Borujeni**: Conceptualization (equal); data curation (equal); formal analysis (equal); investigation (equal); methodology (equal); project administration (equal); resources (equal); supervision (lead); software (equal); validation (equal); visualization (equal); writing—original draft preparation (lead); writing – review and editing (equal). **Amir Hossein Abbasi**: Data curation (equal); investigation (equal); methodology (equal); resources (equal); writing – review and editing (equal). **Mohammad Rahim Haji Hajikolaei**: Conceptualization (equal); data curation (equal); formal analysis (equal); investigation (equal); methodology (equal); project administration (equal); resources (equal); software (equal); validation (equal); visualization (equal); writing – review and editing (equal). **Masoud Reza Seifi Abad Shapouri**: Data curation (equal); investigation (equal); methodology (equal); validation (equal); visualization (equal); writing – review and editing (equal).

## CONFLICT OF INTEREST STATEMENT

The authors declare no conflicts of interest.

### ETHICS STATEMENT

This study was an ‘observational study’ and the research protocol was reviewed and approved by the research committee of the Faculty of Veterinary Medicine, Shahid Chamran University of Ahvaz and documented by the number: 927926. Before beginning work on the present study, we contacted the goat owners, and written informed consent was obtained from the owners for the participation of their animals in this study. During collecting blood specimens, these animals were not disturbed.

### PEER REVIEW

The peer review history for this article is available at https://publons.com/publon/10.1002/vms3.1574.

## Data Availability

The data that support the findings of this study are available from the corresponding author upon reasonable request.
